# The genome sequence of the White Satin, *Leucoma salicis *(Linnaeus, 1758)

**DOI:** 10.12688/wellcomeopenres.19649.1

**Published:** 2023-07-26

**Authors:** Douglas Boyes, Peter W.H. Holland, University of Oxford and Wytham Woods Genome Acquisition Lab, Darwin Tree of Life Barcoding collective, Wellcome Sanger Institute Tree of Life programme, Wellcome Sanger Institute Scientific Operations: DNA Pipelines collective, Tree of Life Core Informatics collective, Darwin Tree of Life Consortium

**Affiliations:** 1 UK Centre for Ecology & Hydrology, Wallingford, England, UK; 2 University of Oxford, Oxford, England, UK

**Keywords:** Leucoma salicis, White Satin, genome sequence, chromosomal, Lepidoptera

## Abstract

We present a genome assembly from an individual male *Leucoma salicis* (the White Satin; Arthropoda; Insecta; Lepidoptera; Erebidae). The genome sequence is 733.2 megabases in span. Most of the assembly is scaffolded into 30 chromosomal pseudomolecules, including the Z sex chromosome. The mitochondrial genome has also been assembled and is 15.73 kilobases in length. Gene annotation of this assembly on Ensembl identified 20,222 protein coding genes.

## Species taxonomy

Eukaryota; Metazoa; Eumetazoa; Bilateria; Protostomia; Ecdysozoa; Panarthropoda; Arthropoda; Mandibulata; Pancrustacea; Hexapoda; Insecta; Dicondylia; Pterygota; Neoptera; Endopterygota; Amphiesmenoptera; Lepidoptera; Glossata; Neolepidoptera; Heteroneura; Ditrysia; Obtectomera; Noctuoidea; Erebidae; Lymantriinae; *Leucoma*; *Leucoma salicis* (Linnaeus, 1758) (NCBI:txid688415).

## Background

Invasive species are organisms that have been introduced outside their normal range by human intervention, accidentally or intentionally, and which then spread ( Simberloff, 2013). Some have the potential to cause harm to the environment, economic activity or human health. Not all introduced species become invasive. In North America, several European species of moth have become invasive, with some causing damage to forests, woodlands and urban trees. An example is the White Satin moth, sometimes called simply the Satin moth, *Leucoma salicis*.

The adult White Satin moth has a silky sheen to the pure white forewings, held tent-like over the body, contrasting with zebra-striped black and white legs. In Britain and Canada, the moth is univoltine with adults on the wing in July and August. After mating, the female lays eggs in clutches of 150–200 on trunks and branches of the food plant. The larvae hatch and feed briefly in autumn, before overwintering and feeding again in spring ( Humphreys, 1996; South, 1961). In Hungary, some populations are bivoltine ( Szöcs *et al.*, 2005). The primary larval food plants are sallow *Salix* sp. and poplar *Populus* sp.

*L. salicis* is native to the Palaearctic with a wide distribution ranging from Portugal and France in the west, through Armenia, Estonia and Russia, to Japan in the east. In Britain, the moth is most common in south-eastern, central and north-western counties of England, with scattered records from Wales and Scotland. There are also records from the Isle of Man and Ireland ( GBIF Secretariat, 2022; South, 1961). In 1920, the species was first reported in North America with specimens caught in Boston, Massachusetts (United States) and in British Columbia (Canada). These were almost certainly the result of accidental introduction. The moth has since spread across the continent and is found in eastern Canada and the north-eastern United States, and on the west coast from British Columbia to Oregon, California and Idaho ( Humphreys, 1996; Latchininsky, no date). In several regions, *L. salicis* is a serious pest. For example, in 2004 large numbers of larvae caused defoliation of hybrid poplar windbreak stands in Wyoming, in 1995 larvae contributed to defoliation of over 6000 hectares of trembling aspen and black cottonwood in the Robson Valley, British Columbia, and in Edmonton *L. salicis* regularly defoliates hybrid poplars planted as ornamental trees along city streets ( Humphreys, 1995; Humphreys, 1996; Latchininsky, no date).

Treatments for pest control have included release of parasitic Hymenoptera and Diptera or spraying with *Bacillus thuringiensis* var *kurstaki* ( Humphreys, 1996). The major female sex pheromone has been identified as (3Z)-cis-6,7-cis-9,10-diepoxy-3-henicosene (‘leucomalure’), with field tests suggesting that different geographic populations may respond to different stereoisomers of the chemical ( Szöcs *et al.*, 2005); pheromone traps have been used for monitoring rather than control.

The genome sequence of *Leucoma salicis* was sequenced as part of the Darwin Tree of Life project. A complete genome sequence for *L. salicis* will facilitate research into pest control strategies, the nature of invasive species, molecular adaptation to host plants and the historical spread of this widespread and economically important species.

## Genome sequence report

**Figure 1.**
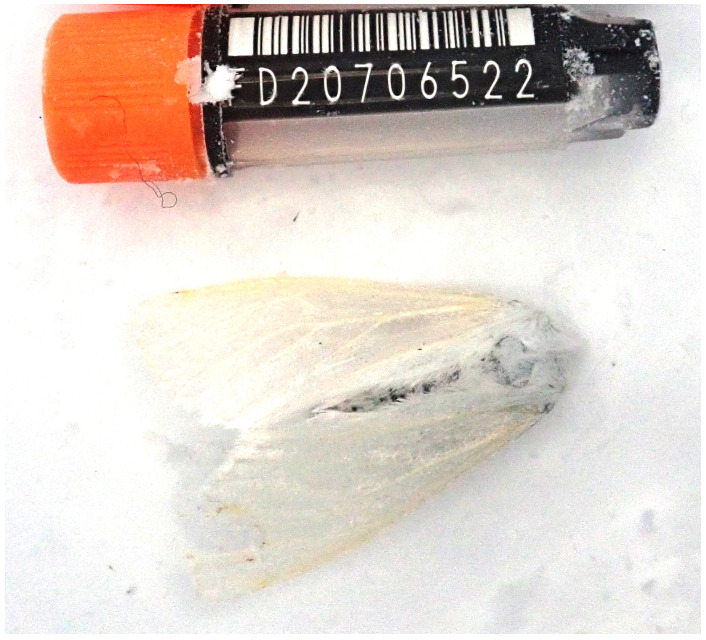
Photograph of the *Leucoma salicis* (ilLeuSali1) specimen used for genome sequencing.

The genome was sequenced from one *Leucoma salicis* ( Figure 1) collected from Wytham Woods, Oxfordshire, UK (51.77, –1.31). A total of 31-fold coverage in Pacific Biosciences single-molecule HiFi long reads was generated. Primary assembly contigs were scaffolded with chromosome conformation Hi-C data. Manual assembly curation corrected 9 missing joins or mis-joins and removed 2 haplotypic duplications, reducing scaffold number by 3.85%.

**Figure 2.**
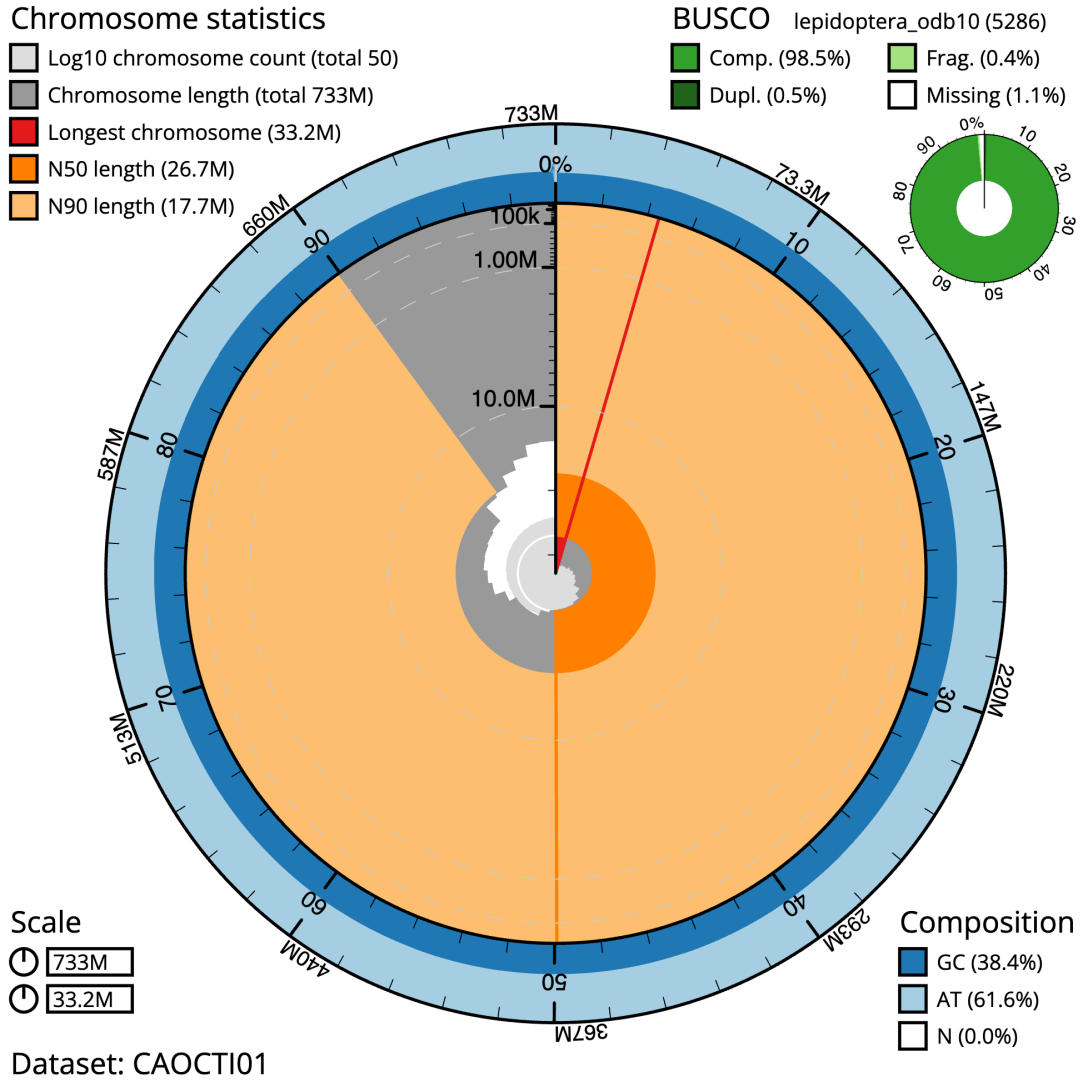
Genome assembly of *Leucoma salicis*, ilLeuSali1.1: metrics. The BlobToolKit Snailplot shows N50 metrics and BUSCO gene completeness. The main plot is divided into 1,000 size-ordered bins around the circumference with each bin representing 0.1% of the 733,224,434 bp assembly. The distribution of scaffold lengths is shown in dark grey with the plot radius scaled to the longest scaffold present in the assembly (33,205,332 bp, shown in red). Orange and pale-orange arcs show the N50 and N90 scaffold lengths (26,724,430 and 17,695,272 bp), respectively. The pale grey spiral shows the cumulative scaffold count on a log scale with white scale lines showing successive orders of magnitude. The blue and pale-blue area around the outside of the plot shows the distribution of GC, AT and N percentages in the same bins as the inner plot. A summary of complete, fragmented, duplicated and missing BUSCO genes in the lepidoptera_odb10 set is shown in the top right. An interactive version of this figure is available at https://blobtoolkit.genomehubs.org/view/ilLeuSali1.1/dataset/CAOCTI01/snail.

The final assembly has a total length of 733.2 Mb in 49 sequence scaffolds with a scaffold N50 of 26.7 Mb ( Table 1). Most (99.84%) of the assembly sequence was assigned to 30 chromosomal-level scaffolds, representing 29 autosomes and the Z sex chromosome. The Z chromosome was identified by synteny to *Euproctis similis* (GCA_905147225.2) ( Boyes & Holland, 2021). Chromosome-scale scaffolds confirmed by the Hi-C data are named in order of size ( Figure 2– Figure 5; Table 2). While not fully phased, the assembly deposited is of one haplotype. Contigs corresponding to the second haplotype have also been deposited. The mitochondrial genome was also assembled and can be found as a contig within the multifasta file of the genome submission.

**Table 1.** Genome data for *Leucoma salicis*, ilLeuSali1.1.

Project accession data		
Assembly identifier	ilLeuSali1.1	
Species	*Leucoma salicis*	
Specimen	ilLeuSali1	
NCBI taxonomy ID	688415	
BioProject	PRJEB58418	
BioSample ID	SAMEA10978935	
Isolate information	ilLeuSali1, male: abdomen (DNA sequencing), head (Hi-C sequencing), thorax (RNA sequencing)	
Assembly metrics *		*Benchmark*
Consensus quality (QV)	67	*≥ 50*
*k*-mer completeness	100%	*≥ 95%*
BUSCO **	C:98.5%[S:98.0%,D:0.5%], F:0.4%,M:1.1%,n:5,286	*C ≥ 95%*
Percentage of assembly mapped to chromosomes	99.84%	*≥ 95%*
Sex chromosomes	Z chromosome	*localised homologous* *pairs*
Organelles	mitochondrial genome assembled	*complete single alleles*
Raw data accessions		
PacificBiosciences SEQUEL II	ERR10704791	
Hi-C Illumina	ERR10684088	
PolyA RNA-Seq Illumina	ERR11242516	
Genome assembly		
Assembly accession	GCA_948253155.1	
*Accession of alternate haplotype*	GCA_948248055.1	
Span (Mb)	733.2	
Number of contigs	147	
Contig N50 length (Mb)	9.2	
Number of scaffolds	49	
Scaffold N50 length (Mb)	26.7	
Longest scaffold (Mb)	33.2	
Genome annotation		
Number of protein-coding genes	20,222	
Number of gene transcripts	20,389	

* Assembly metric benchmarks are adapted from column VGP-2020 of “Table 1: Proposed standards and metrics for defining genome assembly quality” from ( Rhie *et al.*, 2021). ** BUSCO scores based on the lepidoptera_odb10 BUSCO set using v5.3.2. C = complete [S = single copy, D = duplicated], F = fragmented, M = missing, n = number of orthologues in comparison. A full set of BUSCO scores is available at https://blobtoolkit.genomehubs.org/view/ilLeuSali1.1/dataset/CAOCTI01/busco.

**Figure 3.**
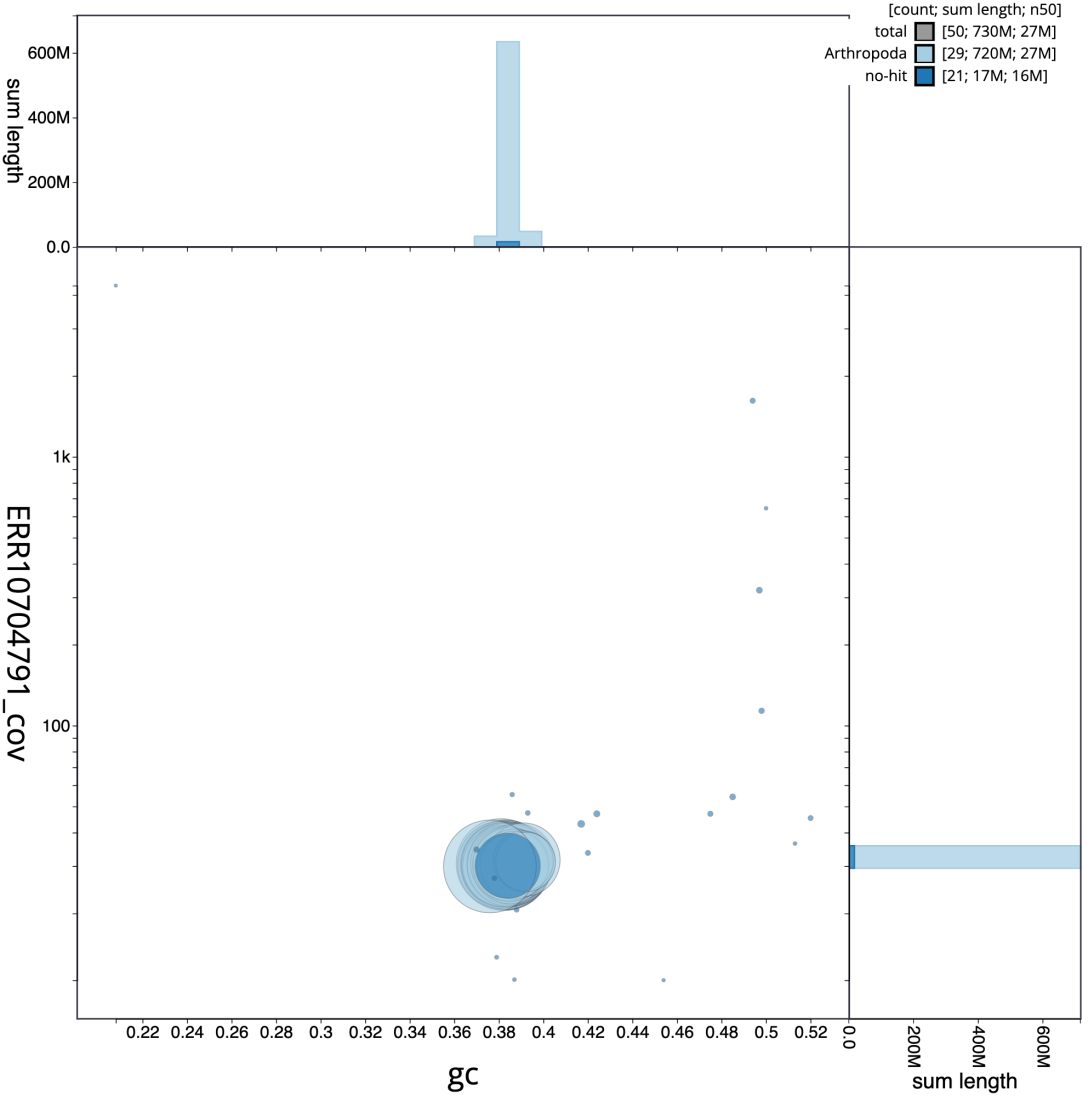
Genome assembly of *Leucoma salicis*, ilLeuSali1.1: BlobToolKit GC-coverage plot. Scaffolds are coloured by phylum. Circles are sized in proportion to scaffold length. Histograms show the distribution of scaffold length sum along each axis. An interactive version of this figure is available at https://blobtoolkit.genomehubs.org/view/ilLeuSali1.1/dataset/CAOCTI01/blob.

**Figure 4.**
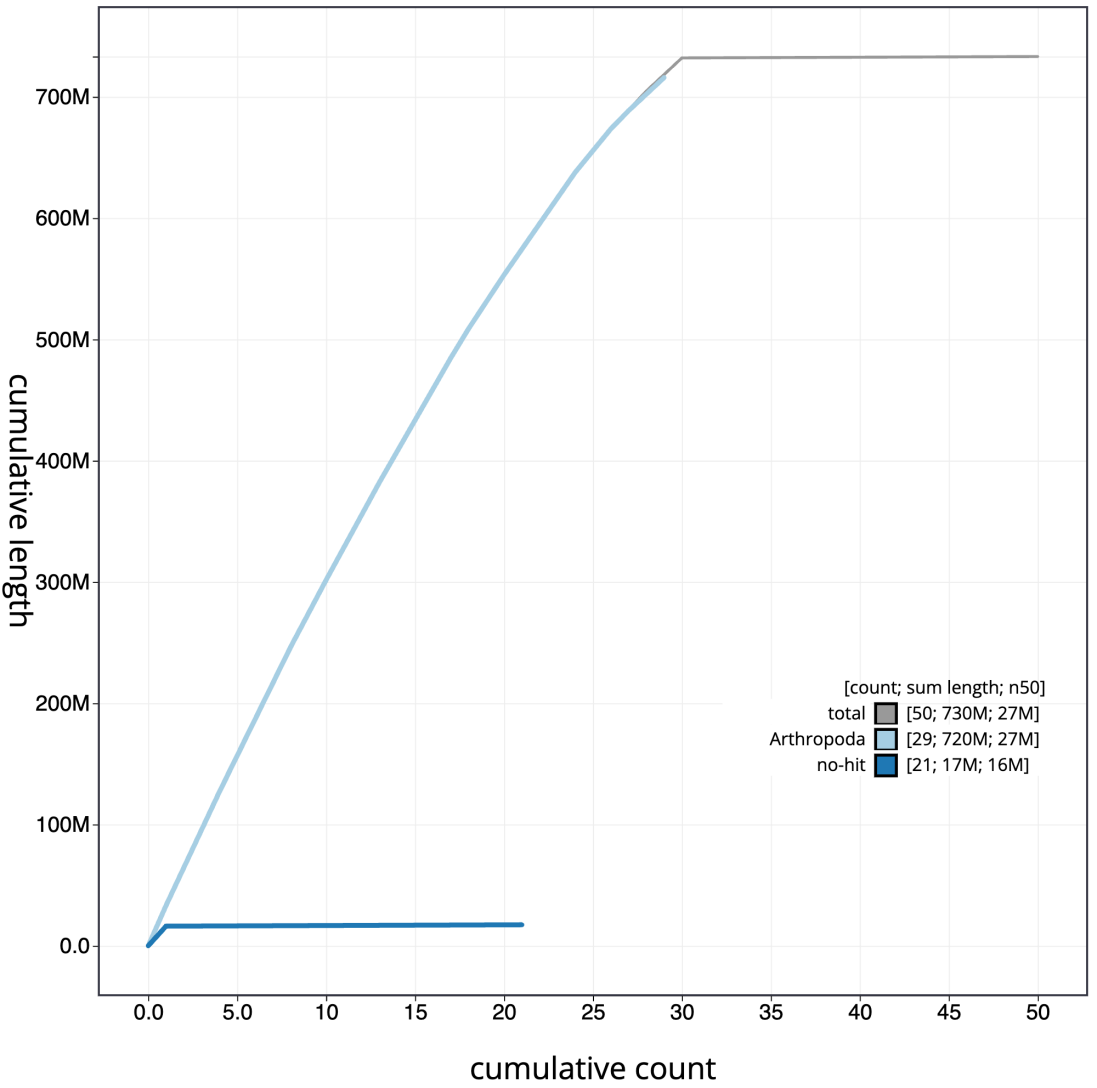
Genome assembly of *Leucoma salicis*, ilLeuSali1.1: BlobToolKit cumulative sequence plot. The grey line shows cumulative length for all scaffolds. Coloured lines show cumulative lengths of scaffolds assigned to each phylum using the buscogenes taxrule. An interactive version of this figure is available at https://blobtoolkit.genomehubs.org/view/ilLeuSali1.1/dataset/CAOCTI01/cumulative.

**Figure 5.**
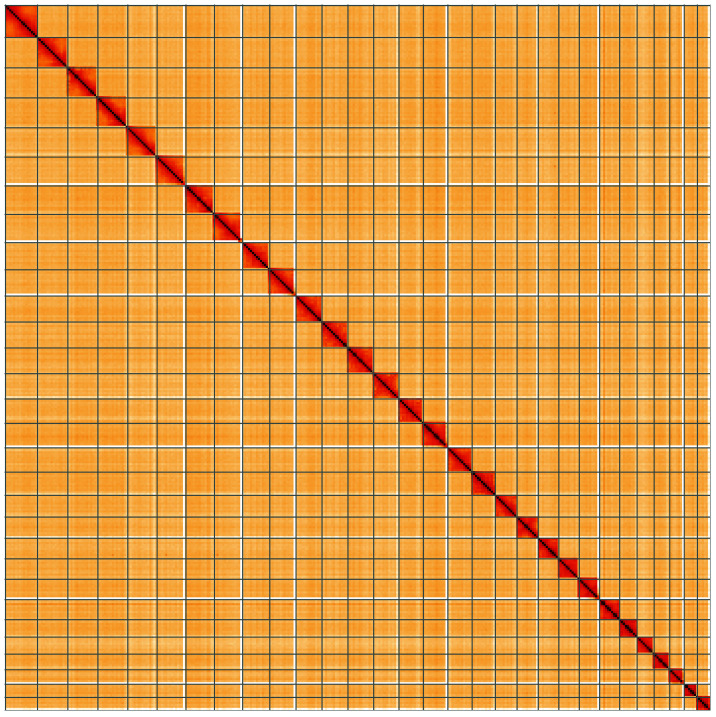
Genome assembly of *Leucoma salicis*, ilLeuSali1.1: Hi-C contact map of the ilLeuSali1.1 assembly, visualised using HiGlass. Chromosomes are shown in order of size from left to right and top to bottom. An interactive version of this figure may be viewed at https://genome-note-higlass.tol.sanger.ac.uk/l/?d=OnIxkXALSL-8Cz4f5BnoPw.

**Table 2.** Chromosomal pseudomolecules in the genome assembly of *Leucoma salicis*, ilLeuSali1.

INSDC accession	Chromosome	Length (Mb)	GC%
OX411804.1	1	31.5	38.5
OX411805.1	2	31.2	38.0
OX411806.1	3	31.04	38.0
OX411807.1	4	30.45	38.5
OX411808.1	5	30.01	38.5
OX411809.1	6	29.59	38.5
OX411810.1	7	29.28	38.0
OX411811.1	8	28.13	38.0
OX411812.1	9	27.26	38.5
OX411813.1	10	26.98	38.5
OX411814.1	11	26.91	38.0
OX411815.1	12	26.72	38.0
OX411816.1	13	26.33	38.5
OX411817.1	14	25.35	38.0
OX411818.1	15	25.32	38.5
OX411819.1	16	25.26	38.5
OX411820.1	17	24.11	38.5
OX411821.1	18	22.53	38.5
OX411822.1	19	21.98	38.5
OX411823.1	20	21.39	39.0
OX411824.1	21	21.27	38.5
OX411825.1	22	21.08	38.5
OX411826.1	23	20.81	39.0
OX411827.1	24	18.11	38.5
OX411828.1	25	17.7	38.5
OX411829.1	26	16.21	38.5
OX411830.1	27	15.04	38.5
OX411831.1	28	13.71	39.0
OX411832.1	29	13.62	39.0
OX411803.1	Z	33.21	37.5
OX411833.1	MT	0.02	21.0

The estimated Quality Value (QV) of the final assembly is 67 with *k*-mer completeness of 100%, and the assembly has a BUSCO v5.3.2 completeness of 98.5% (single = 98.0%, duplicated = 0.5%), using the lepidoptera_odb10 reference set ( *n* = 5,286).

Metadata for specimens, spectral estimates, sequencing runs, contaminants and pre-curation assembly statistics can be found at https://links.tol.sanger.ac.uk/species/688415.

## Genome annotation report

The *Leucoma salicis* genome assembly (GCA_948253155.1) was annotated using the Ensembl rapid annotation pipeline ( Table 1; https://rapid.ensembl.org/Leucoma_salicis_GCA_948253155.1/Info/Index). The resulting annotation includes 20,389 transcribed mRNAs from 20,222 protein-coding genes.

## Methods

### Sample acquisition and nucleic acid extraction

The specimen used for genome sequencing was a male *Leucoma salicis* (specimen ID Ox001666, ilLeuSali1), collected from Wytham Woods, Oxfordshire (biological vice-county Berkshire), UK (latitude 51.77, longitude –1.31) on 2021-07-17 using a light trap. The specimen was collected and identified by Douglas Boyes (University of Oxford), and was snap-frozen on dry ice.

The specimen was prepared for DNA extraction at the Tree of Life laboratory, Wellcome Sanger Institute (WSI). The ilLeuSali1 sample was weighed and dissected on dry ice with tissue set aside for Hi-C sequencing. The tissue was cryogenically disrupted to a fine powder using a Covaris cryoPREP Automated Dry Pulveriser, receiving multiple impacts. DNA was extracted from abdomen tissue of ilLeuSali1 at the WSI Scientific Operations core using the Qiagen MagAttract HMW DNA kit, according to the manufacturer’s instructions.

RNA was extracted from thorax tissue of ilLeuSali1 in the Tree of Life Laboratory at the WSI using TRIzol, according to the manufacturer’s instructions. RNA was then eluted in 50 μl RNAse-free water and its concentration assessed using a Nanodrop spectrophotometer and Qubit Fluorometer using the Qubit RNA Broad-Range (BR) Assay kit. Analysis of the integrity of the RNA was done using Agilent RNA 6000 Pico Kit and Eukaryotic Total RNA assay.

### Sequencing

Pacific Biosciences HiFi circular consensus DNA sequencing libraries were constructed according to the manufacturers’ instructions. Poly(A) RNA-Seq libraries were constructed using the NEB Ultra II RNA Library Prep kit. DNA and RNA sequencing was performed by the Scientific Operations core at the WSI on Pacific Biosciences SEQUEL II (HiFi) and Illumina NovaSeq 6000 (RNA-Seq) instruments. Hi-C data were also generated from head tissue of ilLeuSali1 using the Arima2 kit and sequenced on the Illumina NovaSeq 6000 instrument.

### Genome assembly, curation and evaluation

Assembly was carried out with Hifiasm ( Cheng *et al.*, 2021) and haplotypic duplication was identified and removed with purge_dups ( Guan *et al.*, 2020). The assembly was then scaffolded with Hi-C data ( Rao *et al.*, 2014) using YaHS ( Zhou *et al.*, 2023). The assembly was checked for contamination and corrected as described previously ( Howe *et al.*, 2021). Manual curation was performed using HiGlass ( Kerpedjiev *et al.*, 2018) and Pretext ( Harry, 2022). The mitochondrial genome was assembled using MitoHiFi ( Uliano-Silva *et al.*, 2022), which runs MitoFinder ( Allio *et al.*, 2020) or MITOS ( Bernt *et al.*, 2013) and uses these annotations to select the final mitochondrial contig and to ensure the general quality of the sequence.

A Hi-C map for the final assembly was produced using bwa-mem2 ( Vasimuddin *et al.*, 2019) in the Cooler file format ( Abdennur & Mirny, 2020). To assess the assembly metrics, the *k*-mer completeness and QV consensus quality values were calculated in Merqury ( Rhie *et al.*, 2020). This work was done using Nextflow ( Di Tommaso *et al.*, 2017) DSL2 pipelines “sanger-tol/readmapping” ( Surana *et al.*, 2023a) and “sanger-tol/genomenote” ( Surana *et al.*, 2023b). The genome was analysed within the BlobToolKit environment ( Challis *et al.*, 2020) and BUSCO scores ( Manni *et al.*, 2021; Simão *et al.*, 2015) were calculated.

Table 3 contains a list of relevant software tool versions and sources.

**Table 3.** Software tools: versions and sources.

Software tool	Version	Source
BlobToolKit	4.1.5	https://github.com/blobtoolkit/blobtoolkit
BUSCO	5.3.2	https://gitlab.com/ezlab/busco
Hifiasm	0.16.1-r375	https://github.com/chhylp123/hifiasm
HiGlass	1.11.6	https://github.com/higlass/higlass
Merqury	MerquryFK	https://github.com/thegenemyers/MERQURY.FK
MitoHiFi	2	https://github.com/marcelauliano/MitoHiFi
PretextView	0.2	https://github.com/wtsi-hpag/PretextView
purge_dups	1.2.3	https://github.com/dfguan/purge_dups
sanger-tol/ genomenote	v1.0	https://github.com/sanger-tol/genomenote
sanger-tol/ readmapping	1.1.0	https://github.com/sanger-tol/readmapping/tree/1.1.0
YaHS	1.2a	https://github.com/c-zhou/yahs

### Genome annotation

The BRAKER2 pipeline ( Brůna *et al.*, 2021) was used in the default protein mode to generate annotation for the *Leucoma salicis* assembly (GCA_948253155.1) in Ensembl Rapid Release.

### Wellcome Sanger Institute – Legal and Governance

The materials that have contributed to this genome note have been supplied by a Darwin Tree of Life Partner. The submission of materials by a Darwin Tree of Life Partner is subject to the **‘Darwin Tree of Life Project Sampling Code of Practice’**, which can be found in full on the Darwin Tree of Life website here. By agreeing with and signing up to the Sampling Code of Practice, the Darwin Tree of Life Partner agrees they will meet the legal and ethical requirements and standards set out within this document in respect of all samples acquired for, and supplied to, the Darwin Tree of Life Project. 

Further, the Wellcome Sanger Institute employs a process whereby due diligence is carried out proportionate to the nature of the materials themselves, and the circumstances under which they have been/are to be collected and provided for use. The purpose of this is to address and mitigate any potential legal and/or ethical implications of receipt and use of the materials as part of the research project, and to ensure that in doing so we align with best practice wherever possible. The overarching areas of consideration are:

•   Ethical review of provenance and sourcing of the material

•   Legality of collection, transfer and use (national and international) 

Each transfer of samples is further undertaken according to a Research Collaboration Agreement or Material Transfer Agreement entered into by the Darwin Tree of Life Partner, Genome Research Limited (operating as the Wellcome Sanger Institute), and in some circumstances other Darwin Tree of Life collaborators.

## Data Availability

European Nucleotide Archive: *Leucoma salicis* (white satin). Accession number PRJEB58418; https://identifiers.org/ena.embl/PRJEB58418. ( Wellcome Sanger Institute, 2023) The genome sequence is released openly for reuse. The *Leucoma salicis* genome sequencing initiative is part of the Darwin Tree of Life (DToL) project. All raw sequence data and the assembly have been deposited in INSDC databases. Raw data and assembly accession identifiers are reported in Table 1.

## Author information

Members of the University of Oxford and Wytham Woods Genome Acquisition Lab are listed here: https://doi.org/10.5281/zenodo.4789928.

Members of the Darwin Tree of Life Barcoding collective are listed here: https://doi.org/10.5281/zenodo.4893703.

Members of the Wellcome Sanger Institute Tree of Life programme are listed here: https://doi.org/10.5281/zenodo.4783585.

Members of Wellcome Sanger Institute Scientific Operations: DNA Pipelines collective are listed here: https://doi.org/10.5281/zenodo.4790455.

Members of the Tree of Life Core Informatics collective are listed here: https://doi.org/10.5281/zenodo.5013541.

Members of the Darwin Tree of Life Consortium are listed here: https://doi.org/10.5281/zenodo.4783558.
