# Supplementary material for: The genome sequence of the White Satin, Leucoma salicis (Linnaeus, 1758)
Source: Wellcome Open Res. 2023 Jul 26;8:323. [Version 1] doi: 10.12688/wellcomeopenres.19649.1 (PMC11391186; doi:10.12688/wellcomeopenres.19649.1)
Supplement: Peer review history

# Reviewer report: Reviewer response for version 1

**DOI:** 10.21956/wellcomeopenres.21766.r95982

**Published:** 2024-09-11

**Authors:** Nicol Rueda

**Affiliations:** 1 Wellcome Sanger Institute, Hinxton, England, UK

The article presents a genome assembly from an individual male *Leucoma salicis* (the White Satin; Arthropoda; Insecta; Lepidoptera; Erebidae). In general, the article provides an appropriate background and describes the methods and materials in detail. However, I believe the authors should add the read count and base count for their Hi-C, PacBio, and RNA data. This information could be included in Table 1. Additionally, it would be helpful for readers to know if the genome contains gaps and how many there are.

**A few more recommendations to improve the article:**

 1. In the first paragraph of the Genome Sequence Report, the authors should change "(51.77, –1.31)" to "(latitude 51.77, longitude –1.31)".

 2. Change "protein coding" to "protein-coding".

 3. Replace "Snail plot" with "Snailplot".

Are sufficient details of methods and materials provided to allow replication by others?

Partly

Is the rationale for creating the dataset(s) clearly described?

Yes

Are the datasets clearly presented in a useable and accessible format?

Yes

Are the protocols appropriate and is the work technically sound?

Yes

Reviewer Expertise:

Evolutionary biology, chromosome rearrangements, sex chromosome evolution

I confirm that I have read this submission and believe that I have an appropriate level of expertise to confirm that it is of an acceptable scientific standard.

# Reviewer report: Reviewer response for version 1

**DOI:** 10.21956/wellcomeopenres.21766.r73845

**Published:** 2024-02-19

**Authors:** Cesar J. Benetti

**Affiliations:** 1 Universidad de Len, Len, Spain

This is an interesting paper expanding insect genomic data, especially this species of moth, the white satin *Leucoma salicis* L. (Lepidoptera: Erebidae). The paper presents very complete and detailed information about the genome of the species, the data are reliable, correctly interpreted and represent a very significant contribution to the knowledge of this species and also global insect diversity.

Are sufficient details of methods and materials provided to allow replication by others?

Yes

Is the rationale for creating the dataset(s) clearly described?

Yes

Are the datasets clearly presented in a useable and accessible format?

Yes

Are the protocols appropriate and is the work technically sound?

Yes

Reviewer Expertise:

Insect taxonomy and phylogeny

I confirm that I have read this submission and believe that I have an appropriate level of expertise to confirm that it is of an acceptable scientific standard.
